# Adaptive laboratory evolution enhances cell yield and lipid production of *Chlorella sorokiniana* under mildly cold conditions

**DOI:** 10.3389/fmicb.2025.1715734

**Published:** 2025-11-25

**Authors:** Shu-Yi Lu, Shi-Yu Zhang, Quan-Guo Zhang

**Affiliations:** MOE Key Laboratory for Biodiversity Science and Ecological Engineering, College of Life Sciences, Beijing Normal University, Beijing, China

**Keywords:** artificial selection, lipid accumulation, adaptive laboratory evolution, algal biotechnology, cold adaptation

## Abstract

One challenge to large-scale microalgae cultivation, e.g., for biodiesel production, is the seasonal low-temperature conditions. We argue that seasonally varying selection in natural environments has prevented algae from better adapting to cold temperatures, and that laboratory evolution offers a promising approach for obtaining cold-adapted algal materials. We conducted a population-level artificial selection experiment with the unicellular green microalgae *Chlorella sorokiniana* at both a benign temperature (25 °C) and a mildly cold temperature (15 °C). Four artificial selection regimes were established: random selection, selection for high biomass (i.e., cell yield), selection for high lipid production, and rotation between high-biomass and high-lipid selection. The selection experiment lasted for 12 cycles of culture propagation; cell yield and lipid content were quantified by optical density (OD_750_) measurement and sulpho-phospho-vanillin (SPV) colorimetric method, respectively. We did not observe significant differences among the four selection regimes in evolutionary changes of algal cell yield or lipid yield, suggesting that natural selection at the individual level had dominated the evolutionary changes. Nevertheless, compared with the ancestral strain, selection lines that had evolved at 15 °C typically exhibited increased cell yield and reduced lipid content per cell, indicating a trade-off relationship. However, substantial increases in cell yield may compensate for the reduction in lipid content per cell. Notably, three out of 16 selection lines showed > 1-fold increase in cell yield, and one exhibited >1-fold increase in population-level lipid yield. Selection lines that had evolved at 25 °C exhibited even greater increases in both cell and lipid yields, with a positive relationship between cell yield and lipid content per cell. Our results demonstrated the potential for laboratory evolution to obtain algal materials suitable for biofuel production under seasonal low-temperature conditions.

## 1 Introduction

Microalgae are considered a category of ideal raw materials for biofuel production due to their efficient carbon fixation, rapid growth, strong environmental adaptability, and complete recyclability of biomass (Chisti, 2007; Rawat et al., 2013; Chen et al., 2018; Patle et al., 2021). A large body of research has been devoted to collecting algal materials and identifying culture conditions desirable for faster biomass production and lipid accumulation (Araujo et al., 2011; Sun et al., 2014; Guldhe et al., 2019; Xu and Xiong, 2023; Xu et al., 2024; Zheng et al., 2024). The optimal growth temperature for industrially important microalgae ranges from 20 °C to 30 °C (Ras et al., 2013), hence seasonal low temperatures in non-tropical regions become a hurdle for microalgae cultivation in outdoor environments (Chisti, 2007; Chhandama et al., 2021). Crucially, energy demand is typically higher during low-temperature seasons. Therefore, enhancing algal growth and lipid production under mildly cold conditions (e.g., 10–20 °C) would make a significant contribution to clean energy supply.

Collecting algal materials from natural habitats may not be sufficient for obtaining cold-adapted algae. Natural habitats in non-tropical regions typically show seasonal cycles in temperature. Theory suggests that temporally varying environments usually select for niche generalist genotypes with greater geometric mean of fitness across temporal niches (Kassen and Bell, 1998; Bell, 2007; Jasmin and Kassen, 2007; Kassen, 2014). This leads to an expectation that algae from seasonal environments are unlikely to have been well-adapted to low-temperature conditions. Given the opportunity to constantly evolve under cold conditions, algae are likely to show evolutionary improvement in growth performance.

Indeed, temporally constant selection pressure may be achieved in laboratory environments. The laboratory evolution approach has been used for both answering basic questions in evolutionary biology (Elena and Lenski, 2003; Buckling et al., 2009; Kawecki et al., 2012) and addressing application issues (LaPanse et al., 2021). Examples of the latter included selecting for higher carbon use capacity under elevated CO_2_ conditions (Collins and Bell, 2004; Collins et al., 2006; Li et al., 2015; Jin et al., 2022), and faster carotenoid accumulation under combined blue- and red-light conditions (Fu et al., 2013).

**Figure 1:**
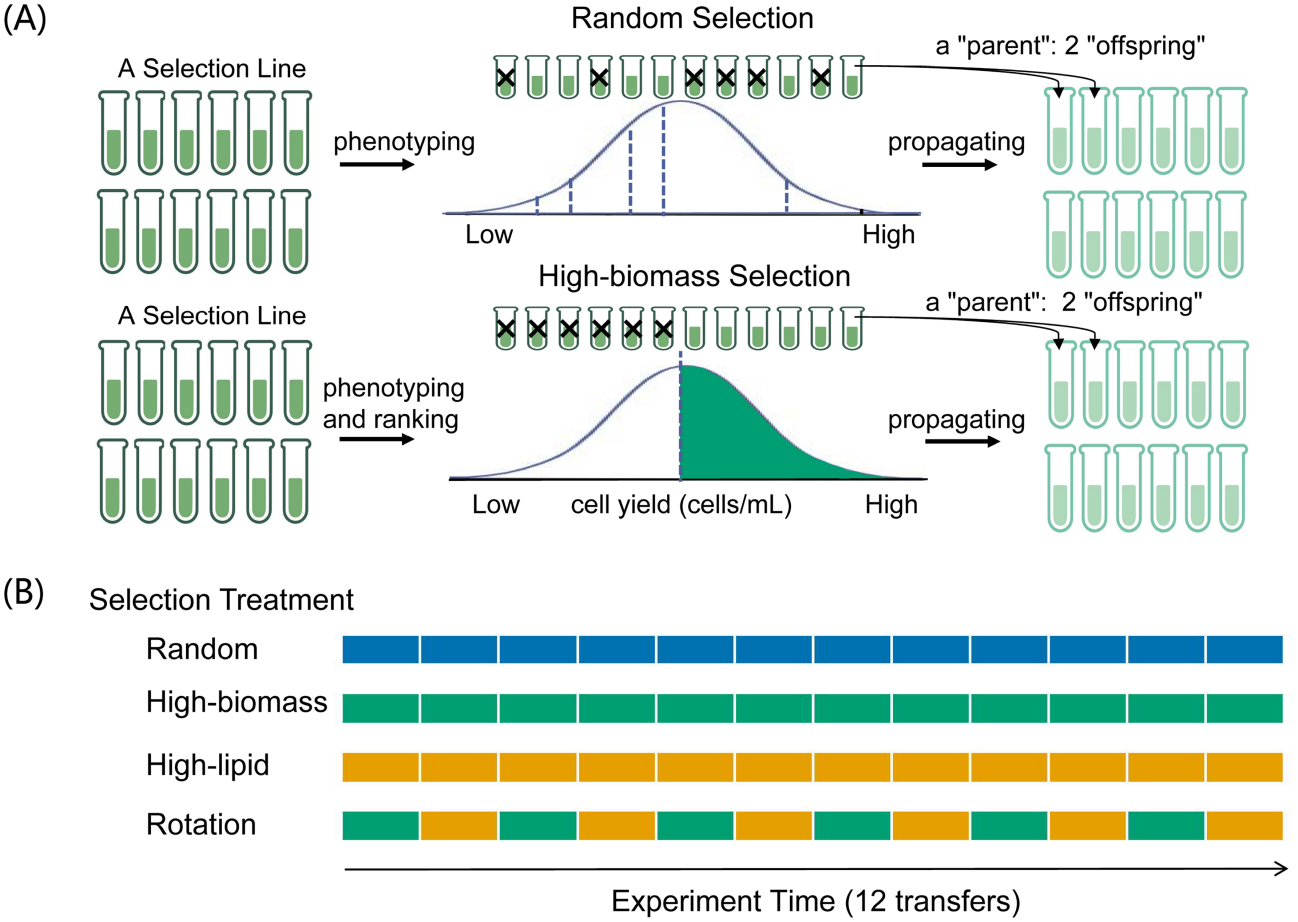
**(A)** A graphical illustration of the artificial selection protocol. Here one cycle of population propagation and selection was illustrated, with random selection and high-biomass selection regimes as examples. Random selection involved choosing six cultures randomly within a selection line (12 cultures) as “parent” populations for the next round of algal propagation, each “parent” culture gave two “offspring” cultures. Under high-biomass selection, the top six cultures in the ranking order of cell yield, measured 1 day before transferring, were chosen to inoculate the next round of algal cultures. Similarly, each “parent” culture gave two “offspring” cultures. **(B)** Four artificial selection regimes involved in the present study. Constant selection (12 transfers) was carried out throughout the experiment under the random, high-biomass, or high-lipid selection regimes, and phases of selection for high- biomass (green) and selection for high- lipid (orange) were alternated under the rotational selection regime.

**Figure 2:**
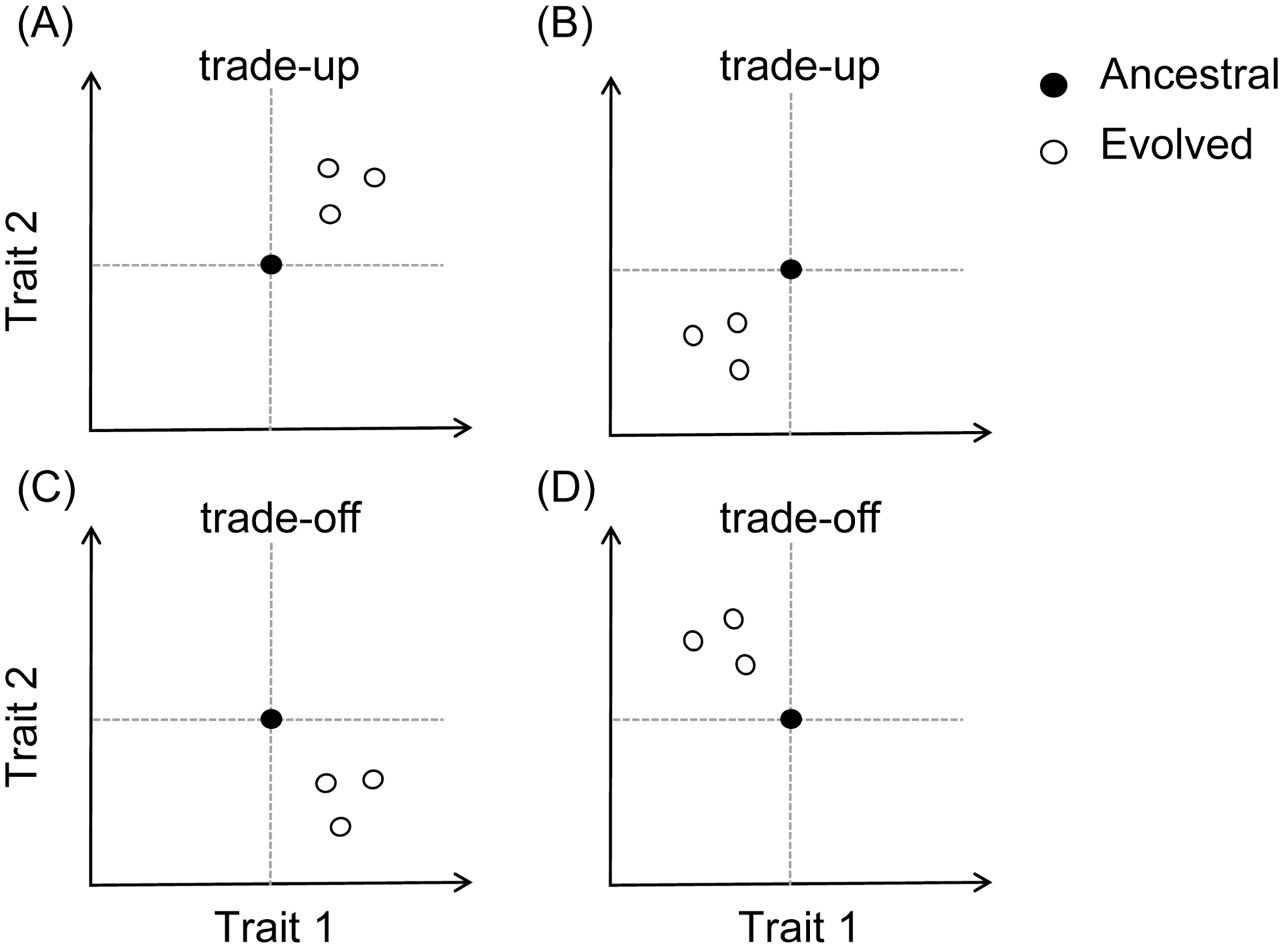
A graphical illustration of evolutionary trade-up **(A,B)** and trade-off **(C,D)** relationships between two traits. During evolutionary adaptation, improvement in one trait may come at a fitness cost to another (trade-off), or both traits may show positively correlated responses to selection (trade-up).

Here we report a laboratory evolution study with *Chlorella sorokiniana*. This unicellular green alga has significant industrial value in biofuel production (Wu et al., 2019). Our study was a population-level artificial selection experiment at two temperatures, 25 °C and 15 °C, the former a benign temperature and the latter a mildly cold temperature. Artificial selection on the population level has been routinely practiced in conventional crop breeding (Murphy et al., 2017; Marín, 2021). Figure 1A graphically illustrates how to perform directional artificial selection, where random selection is a control treatment. In the present study, we considered two target algal traits, cell yield (a proxy of biomass) and lipid yield. During evolutionary adaptation, improvement in one trait may incur fitness costs in the other (trade-off; Figure 2). Alternatively, positively correlated responses to selection may emerge between two traits (trade-up; Figure 2). Both trade-off and trade-up relationships are not uncommon (Barrett et al., 2005; Kassen, 2014). We are particularly interested in the possibility of obtaining algal genotypes with both faster biomass production and greater lipid content. As previous studies have suggested that alternating selection targets may actually accelerate evolutionary adaptation (Kashtan et al., 2007) and may help overcome trade-offs between different traits (Zhang et al., 2021), we considered selection regimes with single target traits, as well as a rotational selection scenario (Figure 1B).

Our study investigated how population-level artificial selection shapes the evolution of algal traits, specifically, the cell and lipid yields, and how evolutionary adaptation may depend on temperatures. Our findings not only address applied challenges in algal biotechnology but also help to understand fundamental questions of evolutionary biology.

## 2 Materials and methods

### 2.1 Culture conditions and methods for trait measurements

The *C. sorokiniana* strain FACHB-24 was purchased from the Freshwater Algae Culture Collection of the Chinese Academy of Sciences (FACHB). Algal cultures were grown in BG11 liquid medium (standard formula; Coolaber, Beijing, China; product no. AML201) under ambient CO_2_ conditions. The batch culture method was used in our experiment. Each culture was propagated in 70 mL of liquid medium in a 100 mL transparent centrifuge tube (with lids loosened to allow for gas exchange), placed in an illumination incubator (GXZ-380A; Ningbo Jiangnan Instrument Factory, China). A light: dark cycle of 12 h:12 h was set, with light intensity of ~57 μmol photons m^−2^ s^−1^. The temperature fluctuation range of the illumination incubator was ±0.2 °C. For every 14 days, 7 mL of each culture was transferred to 63 mL of fresh BG11 medium. The 10-fold population growth during each transfer corresponded to 3.32 generations of binary fission. Cultures were shaken manually once every day, and the positions of the cultures within each incubator layer were alternated daily to minimize environmental heterogeneity among cultures.

**Figure 3:**
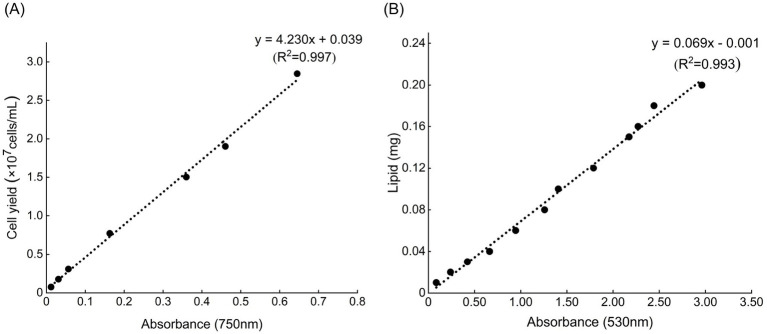
**(A)** The relationship between optical density (OD_750_) and cell yield of *Chlorella sorokiniana*. This relationship was established by making a gradient of algal culture dilutions, and measuring their cell yields using a microscope and OD_750_ using a microplate reader. **(B)** Lipid standard curve was established based on the sulpho-phospho-vanillin (SPV) colorimetric method. Standard lipid stocks were prepared using commercially available soybean oil.

Cell yield was used as a surrogate of biomass. Specifically, 200 μL of sample from each culture was loaded into 96-well plates, and absorbance at 750 nm was measured using a microplate reader (Bio Tek Synergy H1). Each culture was sampled in triplicate for the measurement (technical replicates). The optical density (OD_750_) was then converted to the cell yield. The conversion relationship was as the following: cell yield (10^7^ cells mL^−1^) = 4.230 × OD_750_ + 0.039 (*R*^2^ = 0.997) (Figure 3A). This relationship was established by making a gradient of algal culture dilutions, and measuring their cell yields using a microscope and OD_750_ using a microplate reader.

Lipid content was measured using the sulpho-phospho-vanillin (SPV) colorimetric method (Mishra et al., 2014). Standard lipid stocks were prepared using commercially available soybean oil, and a conversion relationship between lipid content and OD_530_ was established: lipid (mg) = 0.069 × OD_530_–0.001 (*R*^2^ = 0.993) (Figure 3B). A lipid standard curve was prepared by dissolving 100 mg of commercially available soybean oil in 50 mL of a chloroform-methanol (2:1, v/v) mixture, resulting in a stock solution concentration of 2 mg mL^−1^. Aliquots of this stock solution (5, 10, 15, 20, 30, 40, 50, 60, 75, 80, 90, and 100 μL), corresponding to lipid content of 0.01, 0.02, 0.03, 0.04, 0.06, 0.08, 0.10, 0.12, 0.15, 0.16, 0.18, and 0.20 mg, were transferred into separate 15 mL centrifuge tubes. A blank control was prepared without the addition of the stock solution. The organic solvent was evaporated by heating the tubes in a 90 °C water bath for 10 min, followed by the addition of 100 μL deionized water. To measure the lipid content of an algal culture, we centrifuged a 28 mL aliquot for 10 min at 3,176 g (4 °C). After discarding the supernatant, the samples were subjected to the SPV reaction. Briefly, 0.5 mL of concentrated sulfuric acid (98%) was added; then the mixture was heated for 10 min at 90 °C, and cooled in an ice bath for 5 min. Subsequently, 3 mL of freshly prepared phospho-vanillin reagent (1.2 g L^−1^, stored in the dark until use) was added. The reaction was carried out in a shaker at 37 °C (200 rpm) for 10 min. Finally, 200 μL of each sample was transferred to a 96-well plate, and OD_530_ was measured. Each sample was independently measured in triplicate (technical replicates), and the lipid yield (mg L^−1^) was calculated using the lipid standard curve based on algal sample volume.

Under our batch culture conditions, the ancestral algal strain achieved 0.61 ± 0.02 (Mean ± S. E.) × 10^7^ cells mL^−1^ of cell yield and 1.51 ± 0.39 mg L^−1^ of lipid yield at 25 °C. In the 15 °C environment, cell yield was 0.29 ± 0.01 × 10^7^ cells mL^−1^, and lipid yield was 0.55 ± 0.19 mg L^−1^.

### 2.2 The selection experiment

Eight incubators were randomly assigned to 15 °C or 25 °C conditions, four replicates for each temperature. Within each incubator, one replicate selection line for each of the four artificial selection treatments was set up. Thus, we had a total of 32 selection lines: four biological replicate selection lines per artificial selection treatment at each temperature (4 biological replicates × 4 artificial selection treatments × 2 temperatures). Algal cultures founded from a single isolate of *C. sorokiniana* was used to start the experiment. A total of 384 cultures were established with an initial inoculum density of 0.33 × 10^7^ cells mL^−1^ for each culture. These cultures have the same initial genetic composition and physiological state.

Each selection line consisted of 12 individual algal cultures. The four selection treatments were described as follows. (1) Random selection involved choosing six cultures randomly within a selection line as “parent” populations for the next round of algal propagation, each “parent” culture gave two “offspring” cultures (Figure 1A). (2) Under high-biomass selection, the top six cultures in the ranking order of cell yield, measured 1 day before transferring, were chosen to inoculate the next round of algal cultures. Similarly, each “parent” culture gave two “offspring” cultures (Figure 1A). (3) High-lipid selection involved choosing six cultures with greater lipid yield, measured 1 day before transferring. (4) Under the rotation selection, target traits were switched: high-biomass cultures were chosen at transfer 1, 3, 5, etc., and high-lipid cultures were chosen at transfer 2, 4, 6, etc. (Figure 1B).

The selection experiment was conducted for 12 transfers, one transfer every 14 days. During the evolution experiment, we did not observe changes in cell aggregation behavior or cell sizes (Supplementary Figure S1). At the end of the experiment, we measured the cell and lipid yields of each culture at their respective evolutionary temperatures, and the mean values from every selection line were used in subsequent analysis. Selection response in cell or lipid yield was calculated as a log response ratio (LnRR, logarithm of the ratio of evolved culture relative to the ancestral strain).

### 2.3 Data analysis

Data were analyzed in the R environment (version 4.4.2; R Core Team, 2024). At each evolution temperature, one-way ANOVA was used to analyze the difference in selection response in either cell yield or lipid yield among the four selection treatments, and multivariate analysis of variance (MANOVA) was also carried out which considered selection responses in both cell yield and lipid yield as bound response variables. A linear mixed-effects model was used for the effects of experimental evolution temperature and selection treatments on the trait response values, where incubator ID was a random factor. The significance of explanatory variable effects was evaluated using the “Anova” function from the “car” package. One-tailed one-sample *t*-tests were performed to determine whether the mean selection response value was significantly greater than 0. Two-sample *t* tests were employed to analyze the differences between the random-selection control and each of the other three selection treatments (Welch’s correction applied when variances were unequal). Welch’s *t* tests or Wilcoxon test was used to analyze the differences in measured phenotypic values between the ancestor and each selection treatment according to whether the data satisfies the assumptions of normal distribution (Shapiro–Wilk test) and homogeneity of variance (Levene’s test). For multiple comparisons, *p-*values were adjusted using the Benjamini-Hochberg false discovery rate (FDR) method. Effect sizes (Cohen’s *d*) were computed using the package “effsize” to quantify the magnitude of differences between the ancestor and evolved lines. Linear regression analyses were performed to examine the relationships between selection responses in cell yield and those in lipid yield or lipid content per cell under the two temperature regimes. Data visualization was conducted using the “ggplot2” package.

## 3 Results

### 3.1 Selection response and artificial selection regime

**Figure 4:**
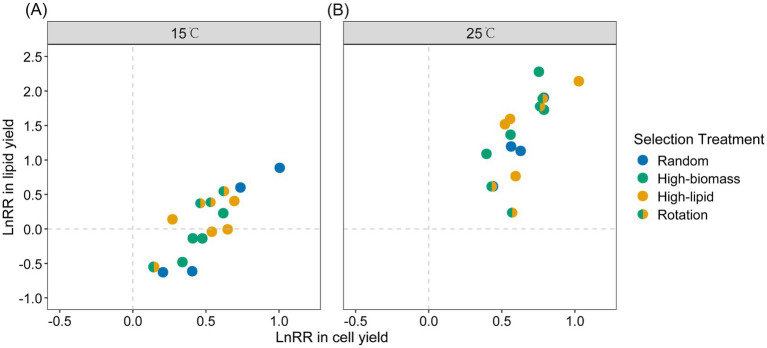
Selection response in cell yield and lipid yield, calculated as the logarithm of the ratio of evolved to ancestral cultures (LnRR). **(A)** Evolution at 15 °C; **(B)** Evolution at 25 °C. Each scatter represents one single selection line (average values across 12 cultures within each selection line). The horizontal and vertical dashed lines through zero indicated the null hypothesis that evolved cultures did not differ from the ancestral strain.

Our selection lines underwent 12 transfers of laboratory evolution. Evolutionary changes in cell yield or lipid yield, termed “selection response,” were calculated as a log response ratio (LnRR, logarithm of the ratio of evolved culture relative to the ancestral strain). A positive LnRR value (LnRR > 0) indicates that the trait value increased during evolution (i.e., the evolved lines exhibited higher cell yield or lipid yield than the ancestor; a positive selection response), whereas a negative LnRR value (LnRR < 0) indicates a decrease in the corresponding trait relative to the ancestor (a negative selection response). Algae from the four selection treatments at 15 °C did not differ in their selection response in cell yield (ANOVA, *F*_3, 12_ = 0.34, *p* = 0.798) or lipid yield (*F*_3, 12_ = 0.28, *p* = 0.839; Figure 4A and see Table 1 for comparisons between the random-selection control treatment and each of the other three selection treatments). MANOVA with selection response in cell yield and lipid yield as bound response variables failed to identify a significant difference among selection regimes either (*F*_6, 24_ = 1.01, *p* = 0.443). Similarly, algae from the 25 °C environment showed no significant difference among selection regimes (Figure 4B, Table 2, and Supplementary Table S1 for ANOVA of selection response in cell yield, *F*_3, 12_ = 0.10, *p* = 0.959; lipid yield, *F*_3, 12_ = 0.57, *p* = 0.647; MANOVA, *F*_6, 24_ = 0.61, *p* = 0.723). These results indicated that there were no significant differences among the four population-level selection regimes in the evolutionary changes of algal cell yield or lipid yield at either evolutionary temperature. The interaction between evolution temperature and artificial selection treatments did not significantly affect the selection responses of cell yield (linear mixed-effects model, *χ^2^_3_* = 1.46, *p =* 0.693) or lipid yield (*χ^2^_3_* = 4.09, *p =* 0.252) (Table 3).

**Table 1:** Summary of the selection response values in the 15 °C selection lines.

Selection treatments	Cell yield		Lipid yield		Lipid content per cell	
	Mean ± S.E.	Difference from random-selection	Mean ± S.E.	Difference from random-selection	Mean ± S.E.	Difference from random-selection
Random	0.59 ± 0.18		0.06 ± 0.40		−0.53 ± 0.20	
High-biomass	0.46 ± 0.06	*df* = 3.67, *p* = 0.535	−0.13 ± 0.14	*df* = 3.78, *p* = 0.673	−0.59 ± 0.07	*df* = 3.85, *p* = 0.806
High-lipid	0.54 ± 0.09	*df* = 4.60, *p* = 0.815	0.12 ± 0.10	*df* = 3.39, *p* = 0.887	−0.41 ± 0.11	*df* = 4.54, *p* = 0.689
Rotation	0.44 ± 0.10	*df* = 4.86, *p* = 0.511	0.17 ± 0.26	*df* = 5.12, *p* = 0.824	−0.27 ± 0.12	*df* = 5.21, *p* = 0.402

Here are shown the Mean (±S.E.) values of selection lines under each selection treatment (*n* = 4). Differences between the random-selection control and each of the other three treatments were analyzed using two-sample *t-*tests.

**Table 2:** Summary of the selection response values in the 25 °C selection lines.

Selection treatments	Cell yield		Lipid yield		Lipid content per cell	
	Mean ± S.E.	Difference from random-selection	Mean ± S.E.	Difference from random-selection	Mean ± S.E.	Difference from random-selection
Random	0.60 ± 0.07		1.21 ± 0.26		0.61 ± 0.17	
High-biomass	0.62 ± 0.09	*df* = 5.70, *p* = 0.874	1.62 ± 0.26	*df* = 5.99, *p* = 0.315	0.99 ± 0.18	*df* = 5.98, *p* = 0.202
High-lipid	0.67 ± 0.12	*df* = 4.97, *p* = 0.636	1.50 ± 0.28	*df* = 5.97, *p* = 0.478	0.83 ± 0.16	*df* = 5.90, *p* = 0.479
Rotation	0.63 ± 0.08	*df* = 5.86, *p* = 0.809	1.12 ± 0.42	*df* = 5.05, *p* = 0.857	0.48 ± 0.28	*df* = 4.68, *p* = 0.773

Here are shown the Mean (± S. E.) values of selection lines under each selection treatment (*n* = 4). Differences between the random-selection control and each of the other three treatments were analyzed using two-sample *t-*tests.

**Table 3:** Linear mixed-effects model results for the phenotypic trait selection response values on cell yield, lipid yield, and lipid content per cell.

Effect type	Term	Cell yield	Lipid yield	Lipid content per cell
Fixed	Evolution temperature	*χ^2^_1_* = 5.30, *p =* 0.021	*χ^2^_1_* = 78.62, *p* < 0.001	*χ^2^_1_* = 96.85, *p* < 0.001
	Selection treatments	*χ^2^_3_* = 1.27, *p* = 0.737	*χ^2^_3_* = 0.99, *p* = 0.803	*χ^2^_3_* = 1.34, *p =* 0.720
	Interaction	*χ^2^_3_* = 1.46, *p =* 0.693	*χ^2^_3_* = 4.09, *p =* 0.252	*χ^2^_3_* = 6.09, *p =* 0.108
Random	Variance (Std. Dev.)	0.021 (0.143)	0.150 (0.387)	0.058 (0.241)
Residual	Variance (Std. Dev.)	0.024 (0.155)	0.173 (0.416)	0.115 (0.339)

Fixed effects include evolution temperature, selection treatments, and their interaction; incubator ID was treated as a random effect. The “Anova” function from the package “car” was used to assess the contribution of each explanatory variable. Chi-squared (χ^2^) statistics with degrees of freedom and *p*-values are reported for fixed effects, and variance components (with standard deviations) are reported for random effects and residuals.

### 3.2 Selection response and temperature

Out of the 16 selection lines that evolved at 15 °C, all exhibited positive values of selection response in cell yield, while eight showed positive selection response values of lipid yield (Figure 4A). Three selection lines showed >1-fold of increase in cell yield compared with the ancestral strain (LnRR > 0.693), and one selection line showed >1-fold of increase in lipid yield. Cultures that had only a small magnitude of evolutionary increase in cell yield typically showed a reduction in lipid yield compared with the ancestral strain (Figure 4A). The mean value of cell yield selection response was 0.51 ± 0.05, significantly greater than zero (one-sample *t-*test, *t* = 9.33, *df* = 15, *p* < 0.001). The 15 °C-evolved selection lines consistently exhibited higher cell yield than the ancestor under 15 °C (Supplementary Figure S2 and Supplementary Table S2), highlighting their substantial adaptive potential to cold conditions. In contrast, the mean value of lipid yield selection response was 0.06 ± 0.12, not significantly different from zero (*t* = 0.49, *df* = 15, *p* = 0.317). These results suggested that evolution at 15 °C mainly favored increased cell yield rather than enhanced lipid production, possibly reflecting a trade-off between resource allocation to growth and lipid storage.

All 16 selection lines that evolved at 25 °C exhibited positive selection responses in both cell and lipid yields (Figure 4B), among which six showed >1-fold increase in both cell yield and lipid yield relative to the ancestral strain. The mean value of cell yield selection response was 0.63 ± 0.04 (one-sample *t-*test, *t* = 15.03, *df* = 15, *p* < 0.001), and the mean value of lipid yield selection response was 1.36 ± 0.15 (*t* = 9.10, *df* = 15, *p* < 0.001). These results indicated that evolution at 25 °C simultaneously enhanced algal growth and lipid accumulation, corresponding to the trade-up scenario shown in Figure 2A, suggesting that higher temperature facilitated coordinated improvements in both traits.

**Figure 5:**
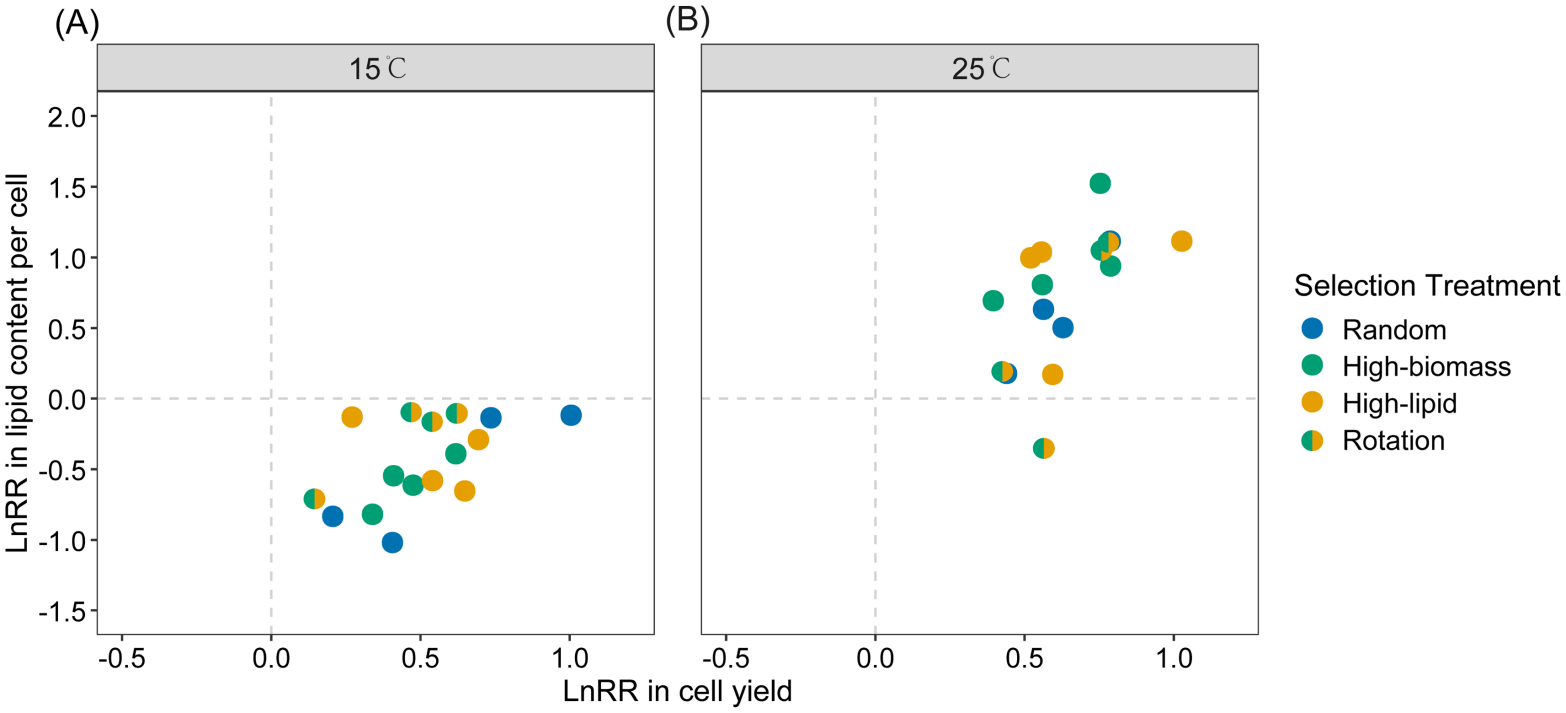
Selection response in cell yield and lipid content per cell, calculated as the logarithm of the ratio of evolved to ancestral cultures (LnRR). **(A)** Evolution at 15 °C; **(B)** Evolution at 25 °C. Each scatter represents one single selection line (average values across 12 cultures within each selection line). The horizontal and vertical dashed lines through zero indicated the null hypothesis that evolved cultures did not differ from the ancestral strain.

Lipid content per cell was also calculated based on cell yield and population-level lipid yield, and its standard error was estimated using bootstrap resampling methods (1,000 iterations). Selection lines that evolved at 15 °C showed a reduction in lipid content per cell compared with the ancestral strain, with a mean value of −0.45 ± 0.08 (one-sample *t*-test *t* = −5.79, *df* = 15, *p* = 1.000; Figure 5A), indicating that adaptation at low temperature did not favor lipid accumulation. By contrast, In the 25 °C environment, algal cultures typically showed significant positive selection response in lipid content per cell, with a mean value of 0.73 ± 0.12 (*t* = 5.99, *df* = 15, *p* < 0.001; Figure 5B), suggesting that higher temperature promoted coordinated improvements in both growth and lipid storage. These results highlighted the role of temperature in modulating the balance between algal growth and lipid accumulation, with potential implications for strain optimization in biofuel production.

## 4 Discussion

It has been suggested that natural selection in temporally varying environments usually favors niche generalists (Rainey and Travisano, 1998; Wittmann et al., 2023; Kumawat et al., 2025). We expected that laboratory evolution of algal cultures under constant temperature conditions would lead to an improvement in growth performance in those specific conditions. The present study showed that laboratory evolution can increase algal performance in both biomass and lipid production, and evolutionary improvement was even greater in the benign environment (Figure 4). On average, at both 15 °C and 25 °C, the cell yield, lipid yield, and lipid content per cell in all evolutionary lines were significantly higher than those of their ancestral strains (Supplementary Table S2). Our results suggested that it pays off for industrial applications of microalgae to use materials specifically adapted to different temperature conditions in different seasons.

Algal cultures from the four population-level selection regimes did not differ in evolutionary changes of algal cell yield or lipid production (Figure 4 and Tables 1, 2). This suggests that natural selection at the individual level has been the predominant force for evolution in our study. Distinct evolutionary changes were nevertheless observed at both temperatures, suggesting that the experimental duration was sufficient for beneficial mutations to arise and reach fixation. Plausibly, there has not been conflicts between population-level traits under artificial selection (cell yield or lipid production) and individual-level fitness (growth traits that determine competitive ability of individuals). In contrast to recent community-level artificial selection studies (Swenson et al., 2000; Xie et al., 2019; Raynaud et al., 2022), our experiment targeted single-species populations and incorporated temperature as a critical environmental selective pressure. This design not only directly addresses the practical challenge of achieving high algal yields under suboptimal temperatures, but also provides direct experimental evidence for how temperature shapes evolutionary responses under artificial selection. We implemented a rotational selection strategy to address multi-trait trade-offs, thereby extending the conventional single-trait selection approach (Garcia et al., 2022; Jacquiod et al., 2022); and we did not observed significant effect of this selection regime.

Algal cultures under both two temperature conditions showed increased cell yield and lipid yield, however, with non-trivial differences. The magnitude of increases in cell and lipid yield was greater under the 25 °C environments (Figure 4). This may be simply because organisms show faster evolutionary adaptation in warmer conditions, as suggested by the evolutionary speed hypothesis (Fischer, 1960; Rohde, 1992; Bennett and Lenski, 1997; Sørensen et al., 2018; Chu et al., 2020). Several mechanisms may contribute to this pattern. Higher temperatures accelerate enzymatic activity and biochemical reactions, promoting faster growth and shorter generation times, which facilitate phenotypic adaptation (Jiang et al., 2016). Increased metabolic rates can also raise mutation frequencies, while larger population sizes in warm conditions strengthen natural selection and reduce the influence of genetic drift, enabling beneficial mutations to fix more efficiently (Jiang et al., 2010; Belfield et al., 2021; McGaughran et al., 2021). Together, these factors may explain why evolutionary improvement in both cell and lipid yields was more pronounced under the benign environment (25 °C).

Meanwhile, temperature may also alter the trade-off relationships among organismal traits. In our algal cultures evolved at 15 °C, a positive selection response in cell yield was accompanied by a reduction in lipid content per cell (Figure 5A), which is corresponding to the trade-off scenario as shown in Figure 2C. This trade-off likely reflects a dilemma in resource re-allocation under cold conditions. Microalgae encounter multiple physiological challenges at low temperatures, including reduced chemical reaction rates, limited enzyme activity, decreased cell membrane fluidity, protein denaturation, and increased water viscosity (Hassan et al., 2016, 2020). Survival under such conditions may be enhanced by the accumulation of major carbon storage compounds such as starch (carbohydrates) and lipids. Starch and lipid metabolism are highly interconnected, sharing glyceraldehyde-3-phosphate (G3P) as a common precursor (Takeshita et al., 2014; Ran et al., 2019). Moreover, the tricarboxylic acid (TCA) cycle and the *de novo* fatty acid synthesis pathway in algae function are competing carbon storage pathways, both utilizing phosphoenolpyruvate (PEP) as a common intermediate metabolite (Zhu et al., 2022). This competition directly affects lipid synthesis and transport. Therefore, cells may prioritize rapid proliferation over energy storage at lower temperatures. Where growth rate is limited by factors other than temperature (e.g., lack of nitrogen, phosphorus, and other nutrient elements), algal cells may instead accumulate lipids at the expense of biomass productivity (Mandal and Mallick, 2011; Ho et al., 2012; Zhu et al., 2022). It is noteworthy that a very large magnitude of increase in cell yield can compensate for reduced lipid content per cell, enhancing population-level lipid production (mg lipid content per L culture; Figure 4A). By contrast, an evolutionary trade-up relationship was observed between cell yield and lipid content per cell in the 25 °C environments, matching the trade-up scenario in Figure 2A. This suggests that the potential conflict in resource re-allocation discussed above may be mitigated under benign temperature conditions. Our findings have practical implications for developing algal strains with optimized biomass and lipid production and for predicting algal performance under varying environmental conditions. Future studies integrating physiological and biochemical analyses will be valuable for deepening our understanding of temperature-dependent resource allocation strategies in microalgae.

Experimental evolution is a promising approach for increasing organism’s tolerance to stresses such as extreme temperature, salinity, and light conditions (Barten et al., 2022), and for enhancing the production of certain metabolites (Fu et al., 2013; Yu et al., 2013; Cho et al., 2024). Here we emphasize that seasonal cold conditions may restrict the industrial applications of microalgae in non-tropical regions, and that laboratory evolution can help to obtain algal materials desirable for biofuel production under mildly cold conditions.

## 5 Conclusion

This study demonstrated that laboratory evolution is a promising approach for obtaining algal materials suitable for biofuel production under mildly cold conditions. Our experiments explored how population-level artificial selection shapes organismal traits and how temperature influences adaptation. Here laboratory evolution showed potential to improve cell yield and lipid production at 15 °C, while more pronounced enhancements were observed at 25 °C. Temperature affected the balance between traits: an evolutionary trade-up (simultaneous improvement of cell yield and lipid content per cell) occurred at 25 °C, whereas a trade-off likely driven by resource allocation constraints was seen at 15 °C. No significant differences were found among the four population-level selection regimes in the evolutionary changes of cell yield or lipid yield, suggesting that natural selection at the individual level was the predominant driver of these changes. These findings highlight the importance of temperature-specific adaptation for bioenergy applications and provide insights into evolutionary mechanisms underlying trait trade-offs in microalgae.

## Data Availability

The raw data presented in this study are publicly available. The data can be found here: https://doi.org/10.6084/m9.figshare.29319644.
