# Supplementary material for: Adaptive laboratory evolution enhances cell yield and lipid production of Chlorella sorokiniana under mildly cold conditions
Source: Front Microbiol. 2025 Nov 25;16:1715734. doi: 10.3389/fmicb.2025.1715734 (PMC12685799; doi:10.3389/fmicb.2025.1715734)
Supplement: Supplementary file 1 [file Table_1.pdf]

Supplementary Material

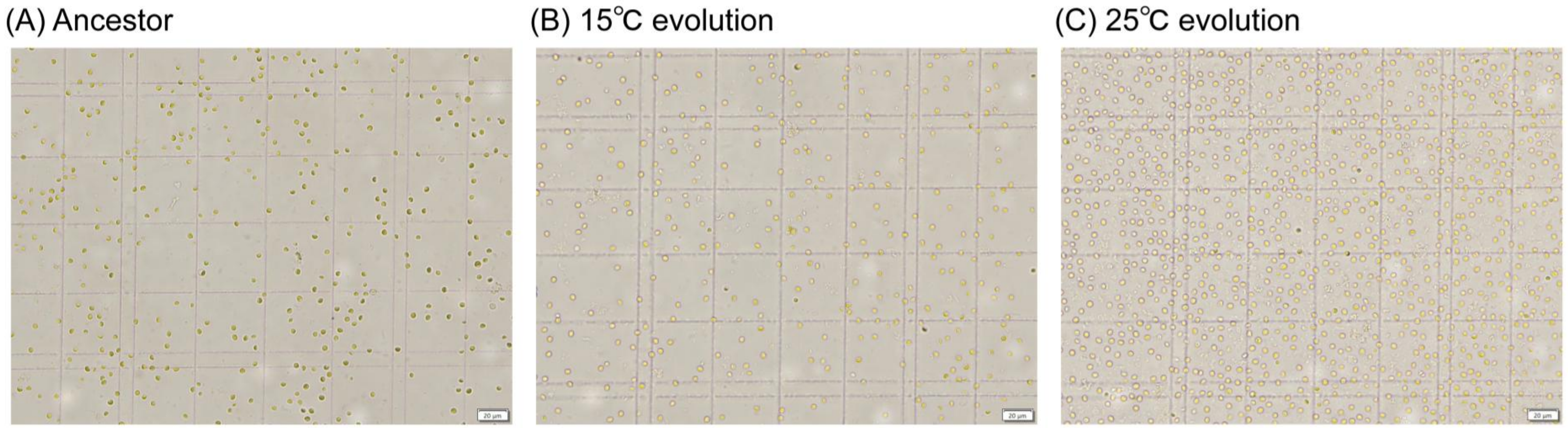

Figure S1 Representative microscopy images of the ancestor and evolved lines at 15 °C and 25 °C (× 400 magnification). No apparent differences in cell morphology or aggregation patterns were observed among the ancestor and populations that evolved at either temperature.

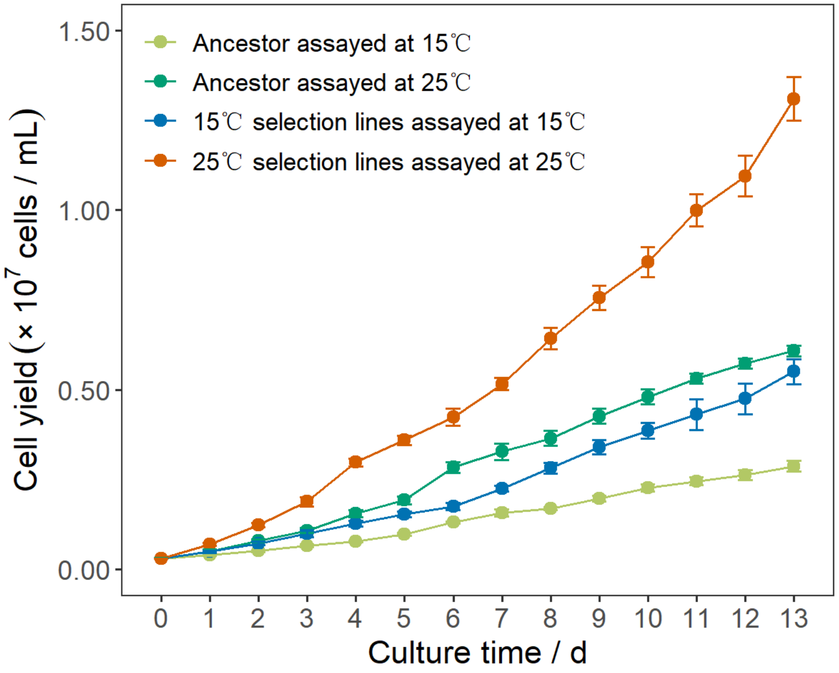

Figure S2 Growth curves of the ancestor and representative evolved lines. Cell yield was monitored over 13 days. One culture was randomly selected from each selection line and assayed at its corresponding evolution temperature (16 biological replicates per temperature). Error bars represent standard errors of replicates. Note that all those assay cultures had a same, relatively low, initial density. Those cultures may not have reach stationary phase during this period of assay. Within-transfer population growth dynamics during selection experiment may not mirror this graph.

Table S1 Summary of the results of one-way ANOVA. Selection responses of cell yield, lipid yield, and lipid content per cell under 15 °C and 25 °C were compared among four selection treatments using one-way ANOVA.

	15 °C	25 °C
Cell yield	$F_{3,12} = 0.34$, $P = 0.798$	$F_{3,12} = 0.10$, $P = 0.959$
Lipid yield	$F_{3,12} = 0.28$, $P = 0.839$	$F_{3,12} = 0.57$, $P = 0.647$
Lipid content per cell	$F_{3,12} = 0.78$, $P = 0.527$	$F_{3,12} = 0.84$, $P = 0.499$

Table S2 Summary of the measured values in the 15 °C and 25 °C selection lines. Here are shown the Mean (± S.E.) values of selection lines under each selection treatment (n = 4). The lipid content per cell was calculated as the ratio of lipid yield to cell yield, and its standard error was estimated using bootstrap resampling methods (1,000 iterations). Differences between the ancestor and each selection treatment were analyzed using Welch's t tests or Wilcoxon test, depending on whether the assumptions of normal distribution and homogeneity of variance were met. Welch's t tests were performed because the response variable cell yield follows a normal distribution but does not meet the assumption of homogeneity of variance. Wilcoxon tests are performed because the response variables lipid yield and lipid content per cell do not meet the assumption conditions. The *P* values were adjusted using the Benjamini–Hochberg false discovery rate (FDR) correction. Effect sizes (Cohen's d) were calculated using the R package "effsize".

	**15 °C**			**25 °C**		
	Mean ± S.E.	Difference from ancestor	Cohen's *d*	Mean ± S.E.	Difference from ancestor	Cohen's *d*
Cell yield (×10^7 cells mL^{-1})						
Ancestor	0.29 ± 0.01			0.61 ± 0.02		
Random	0.55 ± 0.10	*df* = 3.02, *P* = 0.076	-3.18	1.13 ± 0.08	*df* = 3.28, *P* = 0.015	-5.54
High-biomass	0.47 ± 0.03	*df* = 3.19, *P* = 0.019	-5.88	1.16 ± 0.10	*df* = 3.18, *P* = 0.015	-5.16
High-lipid	0.51 ± 0.04	*df* = 3.08, *P* = 0.027	-5.25	1.23 ± 0.16	*df* = 3.07, *P* = 0.030	-4.22
Rotation	0.46 ± 0.04	*df* = 3.08, *P* = 0.038	-4.19	1.16 ± 0.10	*df* = 3.21, *P* = 0.015	-4.45

Mean	0.50 ± 0.03	*df* = 15.94, *P* < 0.001	-2.51	1.17 ± 0.05	*df* = 18.43, *P* < 0.001	-3.59
Lipid yield (mg L^{-1})						
Ancestor	0.55 ± 0.19			1.51 ± 0.39		
Random	0.73 ± 0.26	W = 11, *P* = 0.045	-0.34	5.64 ± 1.56	W = 1, *P* = 0.010	-2.13
High-biomass	0.50 ± 0.07	W = 12, *P* = 0.057	0.11	8.40 ± 2.26	W = 0, *P* = 0.010	-2.61
High-lipid	0.63 ± 0.07	W = 11, *P* = 0.046	-0.18	7.60 ± 1.98	W = 1, *P* = 0.010	-2.59
Rotation	0.71 ± 0.14	W = 10, *P* = 0.037	-0.33	5.85 ± 2.06	W = 4, *P* = 0.048	-1.79
Mean	0.64 ± 0.07	W = 44, *P* = 0.024	-0.24	6.87 ± 0.94	W = 6, *P* < 0.001	-1.70
Lipid content per cell (pg cell^{-1})						
Ancestor	0.19 ± 0.07			0.24 ± 0.05		
Random	0.12 ± 0.05	W = 14, *P* = 0.808	0.42	0.48 ± 0.12	W = 3, *P* = 0.035	-1.45
High-biomass	0.10 ± 0.01	W = 16, *P* = 1.000	0.53	0.70 ± 0.19	W = 0, *P* = 0.010	-2.26
High-lipid	0.13 ± 0.03	W = 15, *P* = 0.933	0.38	0.60 ± 0.16	W = 3, *P* = 0. 035	-2.11
Rotation	0.15 ± 0.02	W = 13, *P* = 0.683	0.25	0.47 ± 0.16	W = 7, *P* = 0.154	-1.17
Mean	0.12 ± 0.02	W = 44, *P* = 0.742	0.57	0.56 ± 0.08	W = 6, *P* = 0.005	-1.50
